# Effect of carnosine synthesis precursors in the diet on jejunal metabolomic profiling and biochemical compounds in slow-growing Korat chicken

**DOI:** 10.1016/j.psj.2023.103123

**Published:** 2023-09-18

**Authors:** Kasarat Promkhun, Chanadda Suwanvichanee, Nathawat Tanpol, Sasikan Katemala, Kanjana Thumanu, Wittawat Molee, Satoshi Kubota, Pekka Uimari, Amonrat Molee

**Affiliations:** ⁎ School of Animal Technology and Innovation, Institute of Agricultural Technology, Suranaree University of Technology, Nakhon Ratchasima 30000, Thailand; † Department of Animal Production Technology, Faculty of Agricultural Technology, Kalasin University, Kalasin 46000, Thailand; ‡ Department of Animal Science, Faculty of Agriculture at Kamphaeng Saen, Kasetsart University, Nakhon Pathom 73140, Thailand; § Synchrotron Light Research Institute (Public Organization), Nakhon Ratchasima 30000, Thailand; # Department of Agricultural Sciences, Faculty of Agriculture and Forestry, University of Helsinki, Helsinki 00790, Finland

**Keywords:** β-alanine, L-histidine, carnosine synthesis, jejunum, metabolite

## Abstract

The slow-growing Korat chicken (**KR**) has been developed to provide an alternative breed for smallholder farmers in Thailand. Carnosine enrichment in the meat can distinguish KR from other chicken breeds. Therefore, our aim was to investigate the effect of enriched carnosine synthesis, obtained by the β-alanine and L-histidine precursor supplementation in the diet, on changes to metabolomic profiles and biochemical compounds in slow-growing KR jejunum tissue. Four hundred 21-day-old female KR chickens were divided into 4 experimental groups: a group with a basal diet, a group with a basal diet supplemented with 1.0% β-alanine, 0.5% L-histidine, and a mix of 1.0% β-alanine and 0.5% L-histidine. The feeding trial lasted 70 d. Ten randomly selected chickens from each group were slaughtered. Metabolic profiles were analyzed using proton nuclear magnetic resonance spectroscopy. In total, 28 metabolites were identified. Significant changes in the concentrations of these metabolites were detected between the groups. Partial least squares discriminant analysis was used to distinguish the metabolites between the experimental groups. Based on the discovered metabolites, 34 potential metabolic pathways showed differentiation between groups, and 8 pathways (with impact values higher than 0.05, *P* < 0.05, and FDR < 0.05) were affected by metabolite content. In addition, biochemical changes were monitored using synchrotron radiation-based Fourier transform infrared microspectroscopy. Supplementation of β-alanine alone in the diet increased the β-sheets and decreased the α-helix content in the amide I region, and supplementation of L-histidine alone in the diet also increased the β-sheets. Furthermore, the relationship between metabolite contents and biochemical compounds were confirmed using principal component analysis (**PCA**). Results from the PCA indicated that β-alanine and L-histidine precursor group was highly positively correlated with amide I, amide II, creatine, tyrosine, valine, isoleucine, and aspartate. These findings can help to understand the relationships and patterns between the spectral and metabolic processes related to carnosine synthesis.

## INTRODUCTION

The Korat chicken (**KR**) is a crossbreed between the Thai indigenous chicken Leung Hang Khao (sires) and the Suranaree University of Technology synthetic breed (dams). KR was developed to provide an alternative breed for the smallholder farmers in Thailand. The main disadvantage of KR is their relatively slow growth rate, and female KR in particular has low performance compared with other commercial breeds (Poompramun et al., 2021). The main advantages of KR are their higher carnosine content, improved water retention ability, and pH_45_ of the meat (Suwanvichanee et al., 2022). Consuming meat with a high content of carnosine has a favorable effect on human health, such as the prevention of diverse age-related diseases (Hipkiss et al., 2013). Besides improving the growth rate of KR, its competitiveness in the Thai chicken industry can be further improved by increasing the carnosine content in KR meat, given that, in the future, consumers may prefer foods that have favorable effects on health.

Carnosine is a cytoplasmic dipeptide synthesized by the bonding of amino acids β-alanine and L-histidine (Perim et al., 2019; Xing et al., 2019; Jukić et al., 2021). It is found at particularly high concentrations in chicken muscles (Jukić et al., 2021). β-Alanine can be obtained through the hepatic breakdown of thymidine, uracil, and dietary dipeptides and is considered a nonproteinogenic rate-limiting precursor of carnosine (Derave et al., 2010). L-histidine is an essential amino acid present in serum and serves as a proteinogenic precursor with bioactive properties (Xing et al., 2019). Catalyzed by the carnosinase enzyme, carnosine degradation occurs in the serum and tissues through hydrolysis before being transmitted to blood vessels by amino transporters (Harris et al., 2012; Sale et al., 2013), and both amino acids can subsequently reassemble as carnosine in the tissue through carnosine synthetase (Blancquaert et al., 2017). In a previous study, β-alanine and L-histidine supplementation in diets enhances carnosine synthesis in the breast muscle without any adverse effect on growth performance, meat quality, and meat texture (Suwanvichanee et al., 2022). Moreover, dietary supplementation with L-histidine alone softens meat toughness in KR chickens (Kubota et al., 2021).

Animals extensively catabolize amino acids in their small intestine. Based on human and mice studies, the jejunum has the highest ability to absorb carnosine compared with other parts of the small intestine because of the high carnosinase enzyme activity of the enterocytes in the jejunal mucosa that act against the carnosine substrate (Sadikali et al., 1975; Ferraris et al., 1988; Sale et al., 2013). However, the metabolic profiling associated with the carnosine synthesis pathway in chickens has not been studied previously. Therefore, in this study, we used untargeted metabolomics and Fourier transform infrared (**SR-FTIR**) microspectroscopy to investigate the metabolic and biochemical changes in the jejunum of KR chickens fed with or without a combination of β-alanine and L-histidine, precursors for carnosine synthesis differentiation, in the diet.

Given that β-alanine and L-histidine are the precursors of carnosine synthesis, our research question is whether different dietary supplementations of the carnosine precursors will provide different mechanisms and/or pathways for the absorption and transportation of the amino acids in the jejunum for carnosine synthesis in meat. Therefore, our main objective is to investigate biochemical compounds, metabolic profiles, and metabolic pathways in the jejunum related to carnosine accumulation in the meat that can be used in the KR chicken breeding program to improve competitiveness in the Thai chicken meat industry.

## MATERIALS AND METHODS

### Ethics Statement

All animal protocols used in this research were approved by the Ethics Committee on Animal Use of the Suranaree University of Technology, Nakhon Ratchasima, Thailand; document ID U1-02631-2559.

### Experimental Design and Sample Collection

We used 40 female KR chickens in this study. Details of the experiment are given in a previous study by Suwanvichanee et al. (2022). Briefly, the experiment was conducted at the experimental farm of the Suranaree University of Technology (**SUT**), Thailand using a total of 400 female KR chickens. Feeding included 3 phases based on chicken age: a starter diet for chickens 1 to 21 d of age (21% protein in diet), a growing phase for chickens 22 to 42 d of age (19% protein in diet), and a finisher diet for chickens 43 to 70 d of age (17% protein in diet). For more details on the composition of experimental feed, see Suwanvichanee et al. (2022). At the age of 28 d, the chickens were divided into 4 experimental groups: a group with a basal diet, a group with a basal diet supplemented with 1.0% (1 g/100 g diet) β-alanine, a group with a basal diet supplemented with 0.5% (0.5 g/100 g diet) L-histidine, and a group with a basal diet supplemented with 1.0% of β-alanine and 0.5% L-histidine. Each experimental group was divided into 5 replicates with 20 chickens. Feed and water were supplied automatically on ad libitum basis. A vaccination program was conducted under recommendation of the Department of Livestock Development, Bangkok, Thailand. The growth performance of chickens, that is, feed intake, body weight gain, average daily feed intake, average daily gain, body weight, and feed conversion ratio, were recorded weekly.

At 70 d of age, 10 randomly selected chickens from each experimental group (2 chickens per replicate) were tagged, stunned by chloroform, exsanguinated by cutting the jugular vein, and allowed to bleed for approximately 2 min. We collected 5 to 10 pieces of 2-cm long whole jejunum segments. For SR-FTIR microspectroscopy measurements, the whole jejunum was cut into 3- to 5-mm thick pieces, washed in a normal saline solution, and stored in 10% buffered formaldehyde. Afterward, the samples were refrigerated for 24 h and transferred to a medium containing 80% ethanol until they were embedded in paraffin wax. In addition, a small amount of the whole jejunum was collected, immediately frozen in liquid nitrogen, and stored at −80°C for metabolomic analysis.

### Prepared Proton Nuclear Magnetic Resonance Spectroscopy (^1^H-NMR) Analyses

Metabolite extraction was performed according to Le Roy et al. (2016). The 40 frozen jejunum samples were thawed at 4°C for 1 h. Then, 0.1 g of tissues were mixed with 1 mL of a 3:1 (v/v) MeOH/H_2_O solution, homogenized for 5 min in a bead beater (BioSpec Products, Inc., Mini-Beadbeater-16, Bartlesvill), and centrifuged at 12,000 × *g* for 10 min at 4°C. The 250 µL of the supernatant extracts were transferred into a 1.5-mL tube, dried in a glass vacuum desiccator for 5 to 6 h at room temperature, and stored at −20°C.

Before ^1^H-NMR analysis, the samples were resuspended in 250 µL of deionized water and 250 μL of 99.9% deuterium oxide (**D_2_O**) (MagniSolv, Darmstadt, Germany). Then, the samples were mixed and 500 uL were transferred into a 5-mm NMR tube (Norwell and BioSpec Products, Inc, Bartlesvill) for the ^1^H-NMR analysis at the Center for Scientific and Technological Eqiupment, Suranaree University of Technology, Thailand. Spectra were collected using a 500-MHz NMR spectrometer (Bruker AVANCE III HD, Karlsruhe, Germany) equipped with a CryoProbe-CPP BBO 500S1. The first increment of a ^2^D-^1^H, ^1^H-nuclear-overhauled effect spectroscopy (**NOESY**) pulse sequence was utilized to acquire ^1^H-NMR data and to suppress the solvent signal. Each spectrum consisted of 32,768 data points collected at 25°C with 128 scans over 16.0 min.

### Metabolite Identification, Quantification, and Pathway Analysis

**Figure 1:**
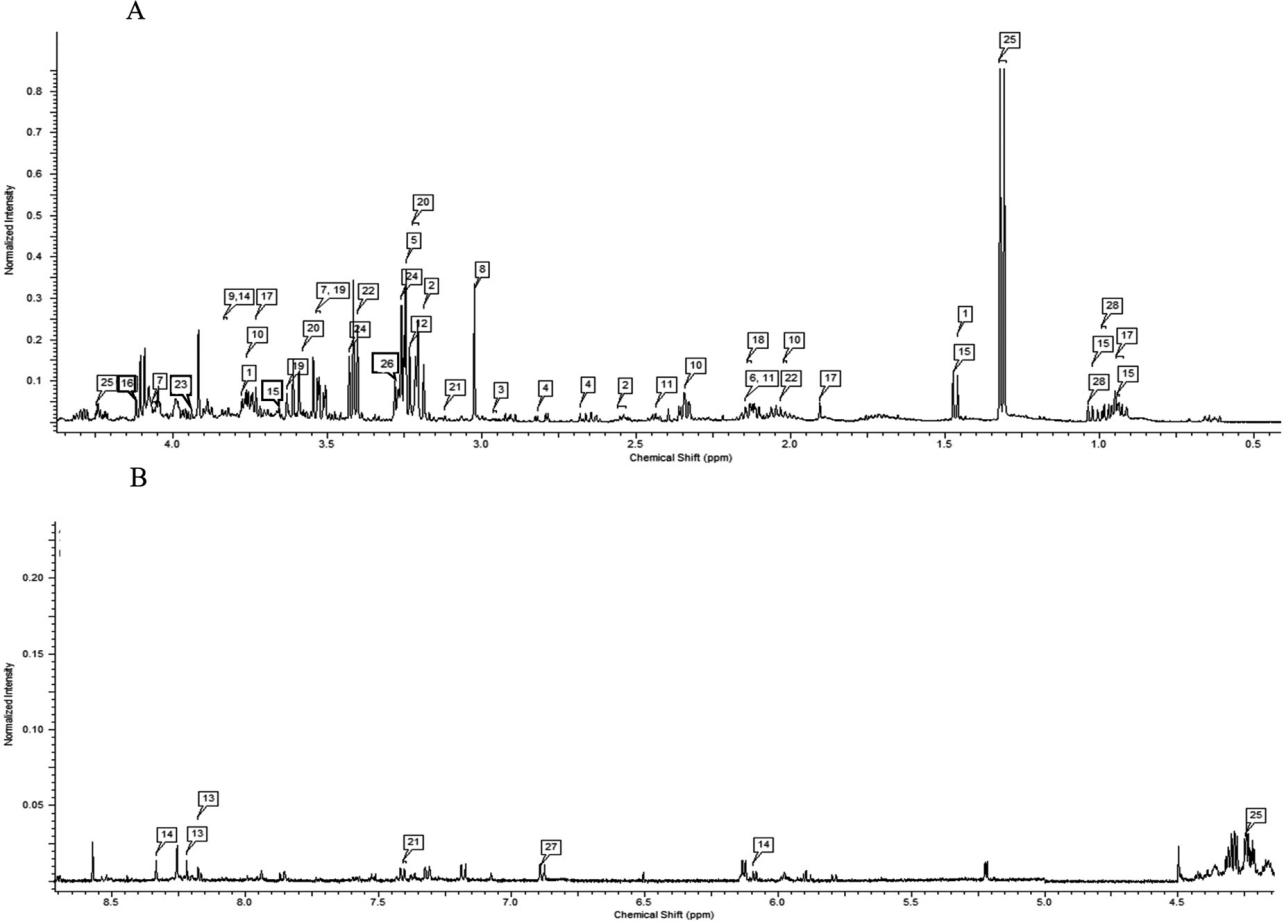
Representation of ^1^H-NMR spectrum at 0.5 to 4.1 ppm chemical shift (δ) region (A) and 4.4 to 8.5 ppm chemical shift (δ) region (B).

Metabolites were determined manually based on chemical shifts (Figure 1, see the labels of the metabolites from Table 1). All points of the free induction decays (**FID**) were Fourier-transformed, and missing values were filled with TOPSPIN software (Version 3.0, Bruker Biospin, Karlsruhe, Germany). The baseline was corrected to the reference resonance at δ = 0.0 ppm with MestReNova 14.2.0-26256 software (Mestrelab Research S.L., Santiago de Compostela, Spain). Spectral regions related to residual water (4.70‒5.10 ppm) were removed (Le Roy et al., 2016) to avoid the effects of imperfect water suppression. In the first step of the process, the 0.5- to 8.5-ppm chemical shift (δ) region was integrated into regions with a width of 0.04 ppm. The data were normalized by the sum over all integral regions using the ACD/NMR predictor. The Chenomx Compound Library was used to compare all the spectra to the internal standard DSS (Xiao et al., 2019).

**Table 1:** Metabolite contents (ppm) in jejunal tissues of the identified metabolites with assigned chemical shifts in the 4 experimental groups.

No.	Metabolites	Chemical shifts (ppm), multiplicity	Experimental groups^1^				*P* value
			A	B	C	D	
1	Alanine	1.46 d, 3.78 q	1417.44^b^	1690.90^a^	1334.72^b^	1497.20^b^	<0.001
2	β-Alanine	2.56 t, 3.19 t	1881.58^c^	2284.48^b^	2378.68^b^	2614.92^a^	<0.001
3	Asparagine	2.96 dd	48.20^bc^	97.30^a^	83.80^ab^	24.86^c^	0.016
4	Aspartate	2.68 dd, 2.82 dd	377.50^a^	391.92^a^	323.44^b^	336.98^b^	<0.001
5	Betaine	3.25 s	8121.36^a^	6554.44^b^	7047.00^b^	6838.52^b^	<0.001
6	Butyrate	2.15 t	417.22^c^	568.84^a^	470.24^b^	542.30^a^	<0.001
7	Choline	3.53 dd, 4.06 t	3188.84^b^	3730.46^a^	3725.10^a^	4004.08^a^	<0.001
8	Creatine	3.02 s, 3.94 s	257.48^c^	314.18^ab^	290.28^b^	342.08^a^	<0.001
9	Ethanolamine	3.13 t, 3.83 t	116.28^c^	140.16^a^	108.68^c^	128.14^b^	0.001
10	Glutamate	2.02 m, 2.34 m, 3.76 dd	300.94^b^	329.00^a^	275.30^c^	328.34^a^	0.009
11	Glutamine	2.15 m, 2.44 m	223.62^b^	271.08^a^	251.62^a^	269.90^a^	<0.001
12	Histidine	3.23 dd, 7.09 s	2727.18^b^	2629.32^bc^	3290.86^a^	2441.92^c^	<0.001
13	Hypoxanthine	8.18 s, 8.21 s	187.1	186.04	182.76	192.18	0.591
14	Inosine	3.83 dd, 4.27 dd, 4.43 dd, 6.09 d, 8.34 s	64.70^ab^	73.74^a^	58.08^ab^	50.54^b^	0.040
15	Isoleucine	0.94 t, 1.02 d, 1.47 dd, 3.65 d	467.24^c^	661.36^a^	561.28^b^	408.68^d^	<0.001
16	Lactate	4.12 q	718.26^b^	908.26^a^	893.36^a^	916.56^a^	<0.001
17	Leucine	0.93 d, 0.94 d, 3.73 m, 1.91 m	1181.88^ab^	1231.94^a^	1104.46^b^	1238.98^a^	<0.001
18	Methionine	2.13 s, 2.14 m	786.44^b^	1083.62^a^	920.88^ab^	1031.26^a^	0.013
19	Myo-inositol	3.53 dd, 3.63 t	1035.82^b^	1235.10^a^	1232.32^a^	1302.42^a^	<0.001
20	Phosphatidylcholine	3.21 s, 3.58 m	357.64^c^	469.58^ab^	480.66^a^	375.38^bc^	0.013
21	Phenylalanine	3.12 dd, 3.26 dd, 7.40 m	65.26	81.38	106.46	81.6	0.299
22	Proline	2.03 m, 3.41 m	503.64^bc^	547.74^a^	462.62^c^	544.54^ab^	<0.001
23	Serine	3.95 dd, 3.95 dd	227.30^ab^	253.80^a^	197.26^b^	201.12^b^	<0.001
24	Taurine	3.43 t	3015.50^a^	2297.58^c^	2601.20^b^	2420.16^bc^	<0.001
25	Threonine	1.32 d, 4.25 m	321.38^a^	253.50^b^	279.58^b^	271.48^b^	<0.001
26	Trimethylamine N-oxide	3.27 s	1217.78	1086.78	1159.7	1189.02	0.730
27	Tyrosine	3.94 dd, 6.89 m	149.38^b^	195.04^a^	122.52^c^	136.86^b^	<0.001
28	Valine	0.98 d, 1.04 d	573.98^b^	681.08^a^	480.40^c^	421.92^d^	<0.001

1 Diet groups. A: control, B: supplementation with 1.0% β-alanine, C: supplementation with 0.5% L-histidine, D: supplementation with 1.0% β-alanine and 0.5% L-histidine. s: singlet, d: doublet, t: triplet, q: quartet, dd: doublet of doublets, and m: multiplet.

a–d Significantly different treatment groups with *P* value <0.05.

Statistical analyses of the modified spectra data of various experimental groups were conducted using the MetaboAnalyst 5.0 platform (http://www.metaboanalyst.ca/). Before statistical analyses, the data were log-transformed and Pareto-scaled (mean-centered and divided by the standard deviation) (Abreu and Fernández, 2020). Data dimensionality was reduced using the supervised partial least squares discriminant analysis (**PLS-DA**), and leave-one-out cross-validation was used for measuring performance accuracy. The coefficient of determination (*R*^2^) and predictive ability (*Q*^2^) were used as initial indicators of the goodness of the model fit. Significance of the metabolites at discriminating the samples was based on the variable importance in the projection (**VIP**) measurement (Xiao et al., 2019); a high VIP value indicates a high contribution of the corresponding metabolite in the discrimination of samples. VIP values greater than 1 were considered significant. For each significant variable, differences between the treatment groups were tested with analysis of variance (1-way ANOVA) using SPSS (SPSS for Windows, version 16.0, SPSS Inc., Chicago, IL). The pair-wise differences between the treatments were tested with the Tukey's HSD post hoc test. Differences with a *P* < 0.05 were considered statistically significant.

The pathway analyses were conducted using the *Gallus gallus* library in the MetaboAnalyst 5.0 platform. Given the exploratory nature of the study, we considered pathways with a *P* < 0.05 to be highly interesting.

### SR-FTIR Microspectroscopy

Paraffin-embedded tissue was washed in 70% ethanol to remove the fixing solutions, dehydrated in graded series of alcohol, cleared in xylol, and embedded into paraffin blocks. Sections were cut with a rotary microtome (HM 340E Electronic, Thermo Fisher, Scientific Microm International GmbH, Walldorf, Germany) into 10-μm thick pieces, mounted onto a barium fluoride (**BaF_2_**) (Crystran Ltd., Dorset, UK) window for spectral acquisition, and dried in a vacuum desiccator before measurement.

The spectra were measured using a Bruker FTIR spectrometer (Vertex70, Bruker Optics, Ettlingen, Germany) coupled with a Bruker Hyperion 2000-IR Microscopy (Bruker Optik GmbH, Ettlingen, Germany) using the 36× IR objective lens with a mercury cadmium telluride (**MCT**) detector cooled with liquid nitrogen over the measurement range from 4,000 to 800 cm^−1^. The transmission mode was used to obtain 64 scans with a 10 × 10 μm aperture size at a resolution of 6 cm^−1^. Spectral acquisition and instrument control were performed using OPUS 7.5 software (Bruker Optics Ltd., Ettlingen, Germany). The background was collected through a blank substrate before spectral analysis. FTIR spectra were measured using synchrotron-based FTIR spectroscopy (BL4.1 Infrared Spectroscopy and Imaging, Synchrotron Light Research Institute, Nakhon Ratchasima, Thailand).

The spectra data were processed with OPUS 7.5 software (Bruker Optics Ltd.). The percentage of integral areas was determined using second-derivative processing from 1,700 to 1,600 cm^–1^ (amide I); 1,600 to 1,500 cm^–1^ (amide II); 1,450 to 1,390 cm^−1^ (CH-bending); 1,320 to 1,220 cm^–1^ (amide III); and 1,200 to 900 cm^–1^ (glycogen and carbohydrate). For the overlapping peaks of the amide I band, the curve fitting based on the Gaussian and Lorentzian functions was applied. The fitting parameters were determined in several regions: β-sheet (1,630 cm^−1^), α-helix (1,644, 1,655 cm^−1^), β-turn (1,670 cm^−1^), and antiparallel (1,689 cm^−1^).

### Principle Component Analysis of SR-FTIR Spectral Data and Metabolomics

The principle component analysis (**PCA**) was applied to cluster and investigate the relationships between the metabolite parameters (with VIP score >1), the biochemical compounds (amide I, amide II, CH-bending, amide III, and glycogen/carbohydrate), and the secondary structure ratios (α-helix, β-sheet, β-turn, and antiparallel) using the Unscrambler X Multivariate Data Analysis software (version 10.1, Camo Analytics, Oslo, Norway). The correlations between the variables were represented by a correlation loading plot. Prior to forming the correlation loading plot, the variables were weighted based on the standard deviation of the variable.

## RESULTS AND DISCUSSION

### Growth Performance Parameter

No significant difference in growth performance was found between the treatment groups. The data on growth performance are published in Suwanvichanee et al. (2022).

### Metabolite Profile Analysis

Based on the ^1^H NMR spectra, 28 metabolites were unambiguously identified in the jejunum (Figure 1). In most cases, the groups fed with carnosine synthesis precursors had higher metabolite levels in the jejunum tissue compared to the control group (Table 1). Based on a previous study by Suwanvichanee et al. (2022), supplementation of both β-alanine and L-histidine, that is, precursors of carnosine synthesis, in the diet (the same diet as in group D) increases carnosine content in the breast meat. In our study, higher concentrations of β-alanine, butyrate, choline, creatine, glutamate, glutamine, lactate, methionine, and myo-inositol but lower concentrations of betaine, threonine, and taurine were obtained in the β-alanine and L-histidine precursor group compared to the control group.

**Figure 2:**
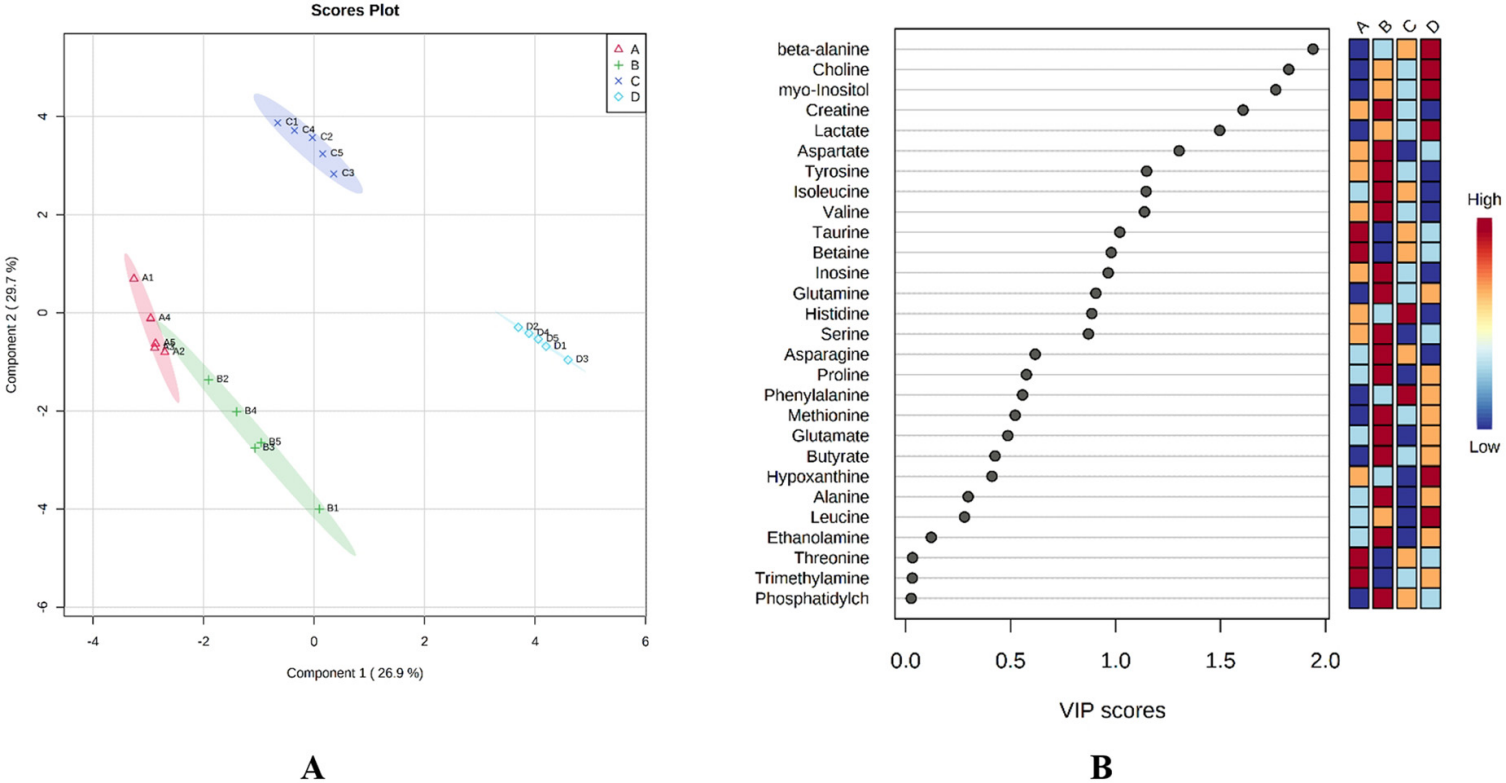
Partial least squares discriminant analysis (PLS-DA) score plot (A) and variable importance in projection (VIP) scores (B) based on jejunum metabolites in the 4 experimental groups; A (control diet), B (diet with 1.0% β-alanine supplementation), C (diet with 0.5% L-histidine supplementation), and D (diet with 1.0% β-alanine and 0.5% L-histidine supplementation).

The experimental groups were clearly separated also based on the PLS-DA score plot (Figure 2A). The first 2 components explained 56.6% of the total variance; component 1 accounted for 26.9% and component 2 accounted for 29.7% of the total variance (Figure 2A). The *R*^2^ = 0.9994 and *Q*^2^ = 0.9933 values demonstrated reasonable qualities for PLS-DA. The results indicate that the metabolite profile of the control group shares similarities with the profile of the β-alanine precursor group, whereas the differences in metabolite profiles between the other groups are noticeable. The most important metabolites for discriminating the treatment groups (VIP >1) were β-alanine, choline, myo-inositol, creatine, lactate, aspartate, tyrosine, isoleucine, valine, and taurine (Figure 2B).

Based on the above results, one of the most interesting findings is the higher level of taurine in the control group compared to groups fed with a diet containing carnosine synthesis precursor β-alanine. Taurine is a nonproteinogenic amino acid, which is abundant and essential for preserving the integrity and function of the eyes, heart, and skeletal muscles in poultry as well as in the small intestine (He et al., 2021). Given that β-alanine is a taurine transporter inhibitor (Murakami and Furuse, 2010), the small intestine will decrease taurine absorption when the diet contains β-alanine, as shown in our study. Likewise, previous studies have indicated that β-alanine reduces the taurine concentration in the breast muscle and plasma of broilers (Qi et al., 2018). A reduction in intracellular taurine may occur as the elevated β-alanine availability increases the competition of their shared taurine transporter (**TauT**) (Shaffer and Kocsis, 1981; Kubo et al., 2022). This may cause a carnosine synthesis increase in KR meat that is supplemented with β-alanine in the diet (Suwanvichanee et al., 2022). Similarly, reduced threonine may be involved in physiological and biochemical processes, including growth functions (Ramos-Pinto et al., 2021). Meanwhile, a decrease in betaine level may be involved in energy metabolism and in support cells to maintain their function during stress periods (Awad et al., 2022). As Suwanvichanee et al. (2022) reported that KR can tolerate stress and may not require carnosine as an antioxidant, we suggest that a decrease of both amino acids (threonine and betaine) has no effect on an antioxidant in modulating the growth performance of KR.

Furthermore, when the diet included both carnosine synthesis precursors, the quantities of β-alanine, choline, myo-inositol, and lactate were increased in the jejunum compared with control group (Figure 2B). Choline is important for metabolomic functions and is absorbed in the jejunum and ileum mainly by an energy- and sodium-dependent carrier mechanism (Smolders et al., 2019). In addition, the initial L-histidine uptake rate is reduced by the substitution of Na^+^ with various forms of choline in the incubation buffer (Sakurai et al., 2002). Choline deficiency usually decreases feed intake and growth rate in poultry (Navidshad et al., 2018). This may be a reason why, in the previous study by Suwanvichanee et al. (2022), no significant difference in growth performance was found between the group supplemented with the carnosine precursor and the control group.

Lactate is a product of the anaerobic metabolism of glucose (Tanaka et al., 2015). Lactase from ingested lactic acid bacteria may enhance the hydrolysis of lactose to glucose and galactose in the small intestine, which is rapidly absorbed or fermented (Hove et al., 1999). Furthermore, an increased myo-inositol concentration in the small intestine, found in all supplementation groups, is associated with increased myo-inositol concentrations in the plasma of broilers (Sommerfeld et al., 2018). Myo-inositol is endogenously synthesized from D-glucose or generated by the dephosphorylation of intracellular myo-inositol hexakisphosphate (Gonzalez-Uarquin et al., 2020b). It is an important molecule in several chicken metabolic processes (Gonzalez-Uarquin et al., 2020a,b) and improves the performance and health of chickens (Lee and Bedford, 2016). Nevertheless, previous studies found that supplementing the carnosine precursor has no effect on production performance in KR (Suwanvichanee et al., 2022). Therefore, these metabolites may play important roles in the overall metabolism involved in carnosine synthesis and may be associated with precursor absorption and transportation in KR jejunum. However, it is important to note that the specific mechanisms of carnosine synthesis, transportation, and utilization in chickens are still an active area of research, and more studies are needed to fully understand the role of carnosine in chickens.

### Metabolic Pathway Analysis

Based on the above results, we expected to identify carnosine synthesis-related metabolic pathways, especially β-alanine and L-histidine metabolisms, as the main metabolic pathways affected by β-alanine and L-histidine supplementation.

**Figure 3:**
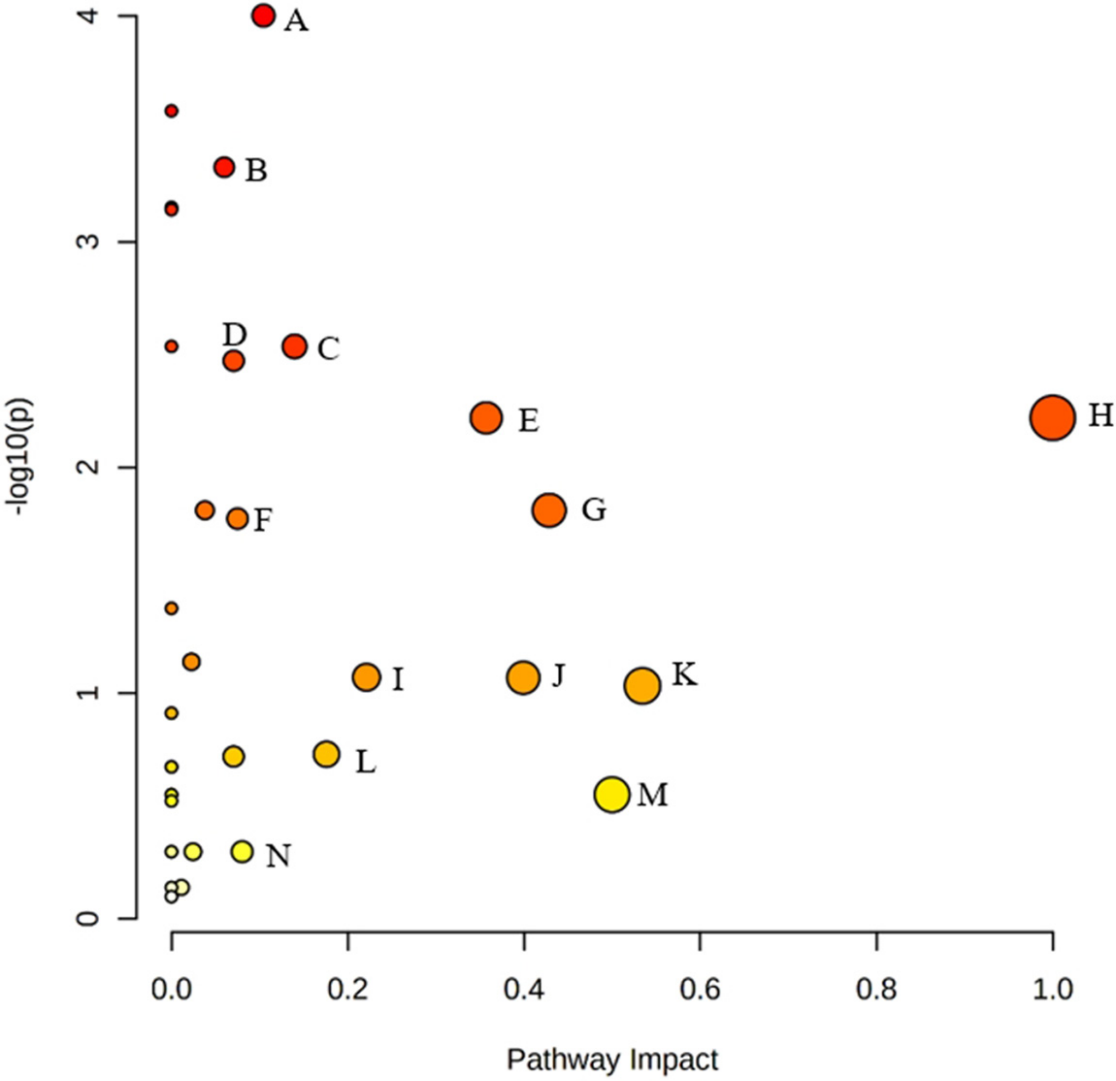
Pathway impact values and *P* values (−log10) from the pathway topology analysis. Color darkness indicates pathway significance (the darker the color the more significant the pathway). (A) Cysteine and methionine metabolism, (B) Glycerophospholipid metabolism, (C) Tyrosine metabolism, (D) Glycine, serine, and threonine metabolism, (E) Phenylalanine metabolism, (F) Pantothenate and CoA biosynthesis, (G) Taurine and hypotaurine metabolism, (H) Phenylalanine, tyrosine, and tryptophan biosynthesis, (I) Histidine metabolism, (J) β-Alanine metabolism, (K) Alanine, aspartate, and glutamate metabolism, (L) Arginine and proline metabolism, (M) D-Glutamine and D-glutamate metabolism, and (N) Inositol phosphate metabolism.

Thirty-four potential metabolic pathways showed differentiation between the groups (Table 2). The results are also shown in the graphical model in Figure 3, where the intensity of the red color indicates the significance of the pathway and the size of the circle corresponds to the pathway impact score. Eight pathways had impact values higher than 0.05, *P* < 0.05, and FDR < 0.05 (Table 2). These pathways were cysteine and methionine metabolism, glycerophospholipid metabolism, tyrosine metabolism, glycine, serine and threonine metabolism, phenylalanine, tyrosine and tryptophan biosynthesis, phenylalanine metabolism, taurine and hypotaurine metabolism, and pantothenate and CoA biosynthesis. In addition, histidine, β-alanine, and alanine, aspartate, and glutamate metabolisms showed high impact values (*P* < 0.05) with *P* values of 0.09.

**Table 2:** List of metabolic pathways showing differentiation between the experimental groups based on the enrichment analysis. *P* values are adjusted for multiple testing. Pathways with *P* value <0.05, FDR < 0.05, and impact >0.05 are marked in boldface.

No.	Metabolic pathway name	Total^1^	Hits^2^	*P* value	FDR^3^	Impact^4^
1	**Cysteine and methionine metabolism**	33	1	0.000099	0.00338	0.10446
2	Valine, leucine, and isoleucine biosynthesis	8	4	0.000263	0.00447	0.00000
3	**Glycerophospholipid metabolism**	35	3	0.000467	0.00489	0.05992
4	Valine, leucine, and isoleucine degradation	40	3	0.000703	0.00489	0.00000
5	Aminoacyl-tRNA biosynthesis	48	13	0.000720	0.00489	0.00000
6	**Tyrosine metabolism**	42	1	0.002907	0.01412	0.13972
7	Ubiquinone and other terpenoid-quinone biosynthesis	9	1	0.002907	0.01412	0.00000
8	**Glycine, serine, and threonine metabolism**	34	4	0.003365	0.01430	0.07063
9	**Phenylalanine, tyrosine, and tryptophan biosynthesis**	4	2	0.006027	0.02049	1.00000
10	**Phenylalanine metabolism**	8	2	0.006027	0.02049	0.35714
11	**Taurine and hypotaurine metabolism**	8	1	0.015473	0.04384	0.42857
12	Primary bile acid biosynthesis	46	1	0.015473	0.04384	0.03799
13	**Pantothenate and CoA biosynthesis**	19	3	0.016845	0.04406	0.07500
14	Butanoate metabolism	15	2	0.042088	0.10221	0.00000
15	Purine metabolism	62	3	0.072628	0.16462	0.02268
16	Histidine metabolism	16	3	0.085022	0.17088	0.22131
17	β-Alanine metabolism	21	3	0.085438	0.17088	0.39925
18	Alanine, aspartate, and glutamate metabolism	28	4	0.092839	0.17536	0.53446
19	Nicotinate and nicotinamide metabolism	15	1	0.122520	0.21925	0.00000
20	Arginine and proline metabolism	38	3	0.186500	0.30901	0.17592
21	Arginine biosynthesis	13	3	0.190860	0.30901	0.07051
22	Glycolysis/gluconeogenesis	26	1	0.212130	0.31359	0.00000
23	Pyruvate metabolism	22	1	0.212130	0.31359	0.00000
24	D-Glutamine and D-glutamate metabolism	6	2	0.281710	0.36839	0.50000
25	Glyoxylate and dicarboxylate metabolism	32	2	0.281710	0.36839	0.00000
26	Nitrogen metabolism	6	2	0.281710	0.36839	0.00000
27	Pyrimidine metabolism	40	2	0.300360	0.37823	0.00000
28	Inositol phosphate metabolism	30	1	0.504150	0.55293	0.08018
29	Phosphatidylinositol signaling system	28	1	0.504150	0.55293	0.02440
30	Galactose metabolism	27	1	0.504150	0.55293	0.00000
31	Ascorbate and aldarate metabolism	10	1	0.504150	0.55293	0.00000
32	Glutathione metabolism	28	1	0.725930	0.74793	0.01123
33	Porphyrin and chlorophyll metabolism	30	1	0.725930	0.74793	0.00000
34	Propanoate metabolism	23	1	0.797300	0.79730	0.00000

1 The total number of metabolites in the pathway was obtained from the library.

2 Number of metabolites actually detected out of 28 metabolites.

3 False discovery rate.

4 The pathway impact value calculated from pathway topology.

Based on previous studies, alanine, aspartate, and glutamate metabolisms are associated with β-alanine and histidine metabolisms and are affected by β-alanine and L-histidine supplementation (Sookoian and Pirola, 2012). In addition, propanoate metabolism, β-alanine metabolism, histidine metabolism, pantothenate and CoA biosynthesis, and alanine metabolism are related to carnosine metabolism (Matthews et al., 2019; Nakata et al., 2020). In chickens, the absorption of carnosine is less efficient than its constituent amino acids L-histidine and β-alanine absorption (Kopec et al., 2020). Previous studies have shown that the breakdown of carnosine into its constituent amino acids occurs rapidly in the small intestine of chickens, with nearly 80% of carnosine being hydrolyzed within 30 min of ingestion (Harris et al., 2012). Therefore, the bioavailability of L-histidine and β-alanine may vary depending on the composition of the diet and the digestive physiology of the chicken.

To conclude, even though the differences between the control and the supplementation groups were not statistically significant but had high impact values, carnosine-related pathways, that is, L-histidine metabolism and β-alanine metabolism, are important in KR jejunum. In addition, we found other pathways involved in β-alanine, L-histidine, and carnosine metabolism, for example, alanine metabolism. The impact of these pathways on metabolic pathways depends on the role of the pathway in the overall metabolism and its function in the organism (Matthews et al., 2019; Nakata et al., 2020).

### The Intensity Ratios of Biomolecules and the Secondary Protein Structure Using SR-FTIR

Synchrotron radiation-based FTIR microspectroscopy techniques were used to confirm the molecular structural and biochemical changes in the jejunum.

**Figure 4:**
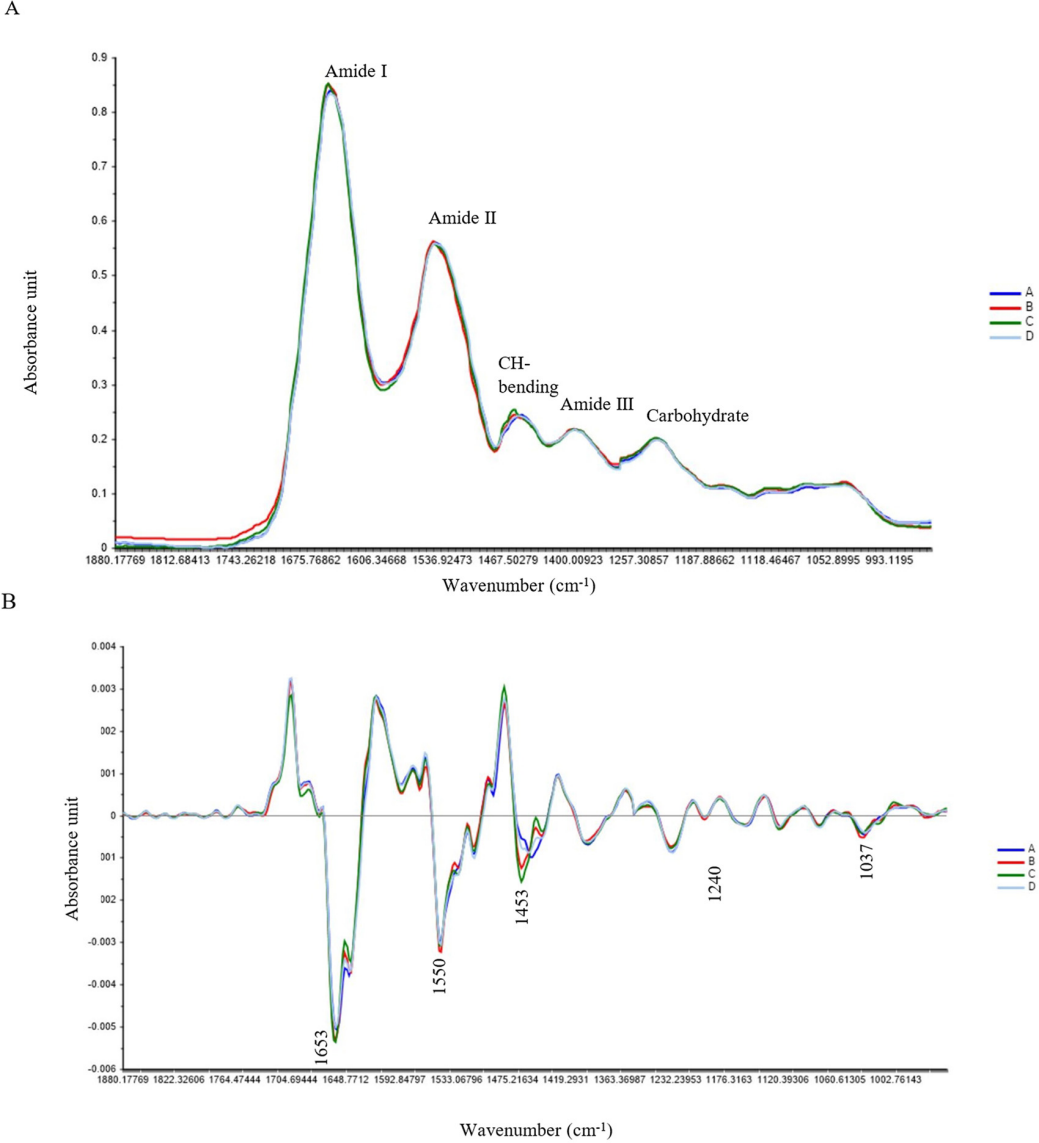
Averages of the original (A) and second-derivative (B) spectra values in the 1,700 to 800 cm^−1^ regions of the experimental groups; A (control diet), B (diet with 1.0% β-alanine supplementation), C (diet with 0.5% L-histidine supplementation), and D (diet with 1.0% β-alanine and 0.5% L-histidine supplementation).

Significant differences in SR-FTIR spectra were obtained in the regions of amide I, II, III, and CH-bending (*P* < 0.05) (Table 3, Figure 4A and B). The integral area of amide I in group B was higher than in the other groups (*P* < 0.05), whereas the control group and L-histidine precursor group did not show significant differences. Related to amide II, the integral areas in group B were higher than in the control group and the β-alanine and L-histidine precursor group (*P* < 0.05) but were not significantly different from the L-histidine precursor group. The β-alanine and L-histidine precursor group showed higher integral areas of CH-bending and amide III than the control, β-alanine, and L-histidine precursor groups (*P* < 0.05). Regarding CH-bending, the integral areas of the L-histidine precursor group were lower than in the control group, but the β-alanine precursor group was not significantly different from the control group. In addition, glycogen/carbohydrate showed nonsignificant differences among 4 groups (*P* > 0.05).

**Table 3:** Integral areas of the SR-FTIR spectra averaged over 200 spectra per experimental group.

Biomolecule (wavenumber)	Experimental groups^1^					
	A	B	C	D	SEM^2^	*P* value
Amide I (1,700–1,600 cm^−1^)	0.0070^b^	0.0080^a^	0.0070^b^	0.0064^c^	0.0001	<0.001
Amide II (1,600–1,500 cm^−1^)	0.0020^b^	0.0034^a^	0.0026^ab^	0.0022^b^	0.0003	0.007
CH-bending (1,450–1,390 cm^−1^)	0.0008^b^	0.00001^c^	0.0010^b^	0.0038^a^	0.0002	<0.001
Amide III (1,320–1,220 cm^−1^)	0.0010^b^	0.0010^b^	0.0006^b^	0.0032^a^	0.0002	<0.001
Glycogen/carbohydrate (1,200–900 cm^−1^)	0.0012	0.0020	0.0016	0.0016	0.0005	0.450

1 Diet groups. A: control, B: supplementation with 1.0% β-alanine, C: supplementation with 0.5% L-histidine, D: supplementation with 1.0% β-alanine and 0.5% L-histidine.

2 Standard error of mean.

a–c Significantly different treatment groups with *P* value <0.05.

Our results suggest that carnosine synthesis precursors strongly affect amide I, II, and III and CH-bending in the chicken jejunum. Previous studies (Yu and Nuez-Ortín, 2010) indicate that the amide I to amide II ratio has a positive correlation with the metabolizable protein, while the slowly degradable fraction of protein is positively correlated with the amide I level. The amide I band, which is sensitive to small differentiation in molecular structure and hydrogen bonding motifs, is important for determining protein structural and conformational changes (Ismael et al., 2019). Therefore, it is possible that the group of β-alanine precursor may affect amide I protein transformation because amide I can provide information on the vibrational bands of the protein backbone (Sadat and Joye, 2020). Moreover, amide III levels were high in the group of β-alanine and L-histidine precursors, characterized by changes in collagen secondary structures and CH-bending (Dehring et al., 2006). The CH-bending in β-alanine and L-histidine refers to the movement of the hydrogen atoms attached to the carbon atoms in these amino acids and contains CH bonds that can undergo bending motions (Freire et al., 2017). Likewise, CH-bending in β-alanine and in carnosine can have important implications to the biological activity of these molecules and to their interactions with other molecules in the body (Matthews et al., 2019). Accordingly, CH-bending was found to be high in the β-alanine precursor group.

Moreover, to determine the secondary structure of proteins, we calculated second derivatives of the obtained FTIR spectra and made a deconvolution of amide I at the IR region. The proportion of different protein secondary structures in the amide I region is presented in Table 4. Significant differences between the experimental groups were obtained for the β-sheet and α-helix (*P* < 0.05). The content of β-sheet in the β-alanine precursor group and in the L-histidine precursor group was higher than in the control group or in the β-alanine and L-histidine precursor group. In addition, the α-helix content in the β-alanine precursor group was lower than that in the control group.

**Table 4:** Relative proportion (%) of secondary protein structures in the amide I region averaged over 200 spectra per experimental group.

Secondary protein structure (wavenumber)	Experimental group^1^				SEM^2^	*P* value
	A	B	C	D		
β-Sheet (1,630 cm^−1^)	25.12^b^	27.06^a^	27.21^a^	25.51^b^	0.421	<0.001
α-Helix (1,644 cm^−1^, 1,655 cm^−1^)	34.75^a^	32.19^b^	33.27^ab^	33.67^ab^	0.621	0.007
β-Turn (1,670 cm^−1^)	18.95	18.15	18.38	18.95	0.497	0.296
Antiparallel (1,689 cm^−1^)	21.18	22.60	21.14	21.86	0.661	0.132

1 Diet groups. A: control, B: supplementation with 1.0% β-alanine, C: supplementation with 0.5% L-histidine, D: supplementation with 1.0% β-alanine and 0.5% L-histidine.

2 Standard error of mean.

a,b Significantly different treatment groups with *P* value <0.05.

Our results confirmed that carnosine synthesis precursor supplements in the diet influence amide I and protein secondary structures in the jejunum; namely the β-alanine precursor group increases the β-sheet and decreases the α-helix content in chicken jejunum. β-Alanine precursor supplementation in the diet has also been shown to have a beneficial effect and to diminish tissue damage in the small intestine in rats (Brencher et al., 2017). In addition, elevating the β-sheet to an α-helix ratio can increase the digestible and indigestible protein fraction in animals, and a high percentage of the β-sheet in amide I may cause low access to digestive enzymes in the gastrointestinal tract (Yu, 2004).

Consistent with previous studies by Abbasi et al. (2014), Cui et al., 2020, and Qiu et al. (2022), we have shown that carnosine precursor supplementation in the diet may affect the biochemical compounds in the jejunum.

### PCA Analysis of Biochemical Compounds, Protein Secondary Structures, and Metabolite Content

**Figure 5:**
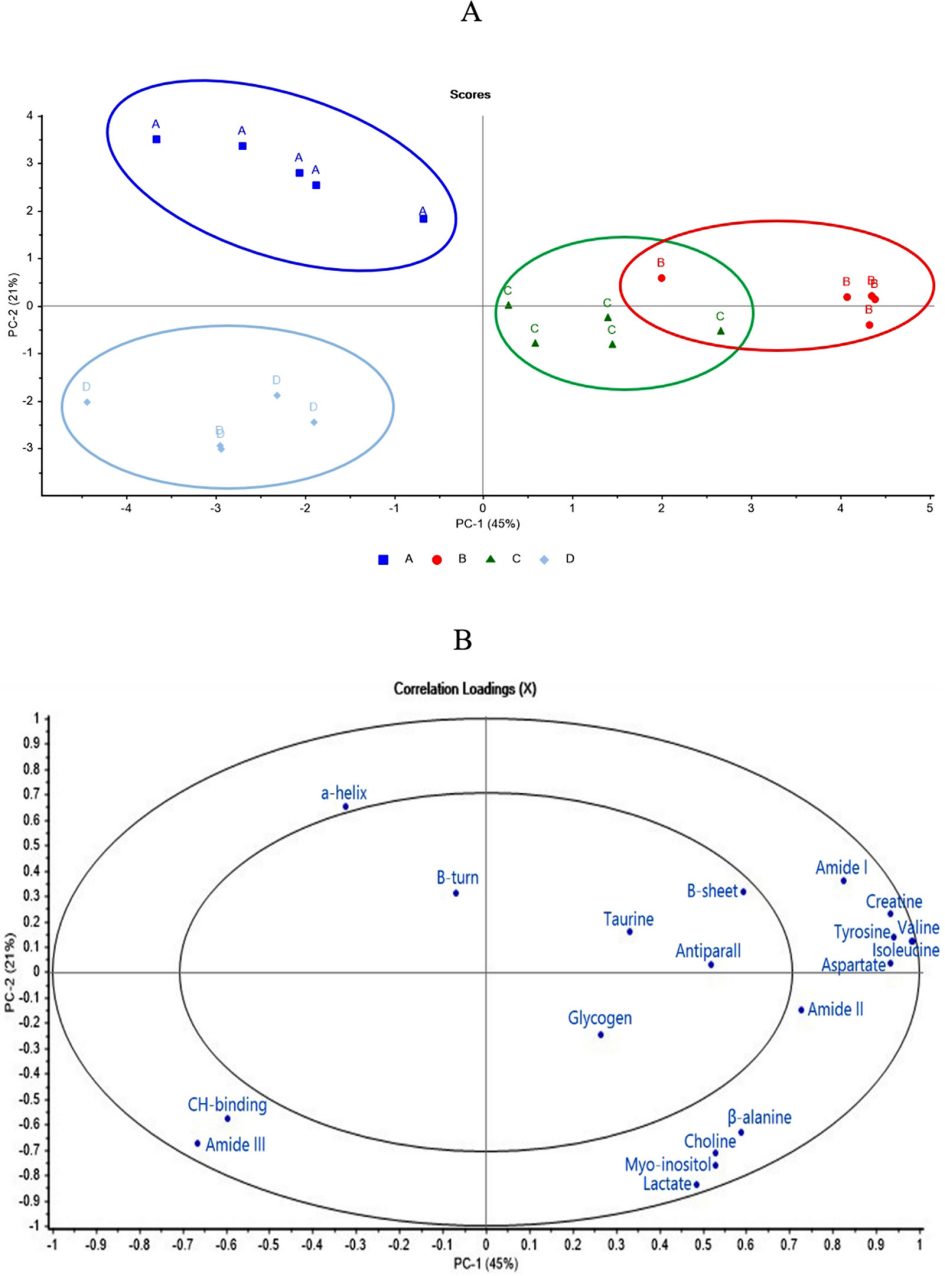
Principal component (PC) analysis score plot (A) for PC1 vs. PC2 of the different experimental groups and correlation loading plot (B) for PC1 vs. PC2. The PCs explain 66% of the total variance. Experimental groups; A (control diet), B (diet with 1.0% β-alanine supplementation), C (diet with 0.5% L-histidine supplementation), and D (diet with 1.0% β-alanine and 0.5% L-histidine supplementation).

Principal component analysis was used to confirm the dependence between the metabolites that had a VIP value >1 and both the biochemical compounds and protein secondary structures. The score plot and correlation loadings related to SR-FTIR spectra, secondary structure, and metabolite content are shown in Figure 5A and B. These plots show that the first 2 principal components (**PCs**) carry a large amount of information, explaining 66% of the data variance, with PC1 explaining the most (45%) and PC2 explaining much less variance (21%). The plot contains 2 ellipses that indicate how much variance is considered; the outer ellipse indicates 100% of the explained variance and the inner ellipse indicates 50% of the explained variance. Variables that are found between the 2 ellipses, and particularly those positioned near the edge of the outer ellipse, are those that are most important in the differentiation. All variables located in the outer circle region (α-helix, amide I, II, III, CH-bending, creatine, tyrosine, valine, isoleucine, aspartate, β-alanine, choline, myo-inositol, and lactate) were significantly correlated with different experimental groups; the control group was positively correlated with α-helix, while the β-alanine precursor group, and the L-histidine precursor group were highly positively correlated with amide I, II, creatine, tyrosine, valine, isoleucine, and aspartate, and the β-alanine and L-histidine precursor group was positively correlated with amide III and CH-bending.

The results indicate signaling or metabolic pathways that are involved in the absorption and transportation of carnosine synthesis precursors. Overall, these metabolites can be used for determining the biochemical compound and protein structure changes and for monitoring precursor responses in the jejunum.

## CONCLUSIONS

In this study, we investigated the metabolic and biochemical compound changes in KR jejunum caused by β-alanine and L-histidine supplementation in the diet. We identified metabolite contents and metabolic pathways that could be linked to these amino acid precursors. The 10 key metabolites that differentiated the diet groups were β-alanine, choline, myo-inositol, creatine, lactate, aspartate, tyrosine, isoleucine, valine, and taurine. The relative proportion of different secondary protein structures indicated that the β-alanine precursor in the diet increases β-sheets and decreases α-helix in the amide I region in jejunal tissue. In addition, an L-histidine precursor in the diet increases β-sheets in the amide I region. PCA revealed that a diet with both precursors of the carnosine synthesis is strongly and positively associated with amide I, amide II, creatine, tyrosine, valine, isoleucine, and aspartate levels in the jejunum. Therefore, our study confirmed that precursor supplements in the diet influence biochemical changes in jejunal tissue and can be used for monitoring the precursor response. However, a deeper understanding of the interplay between metabolites and transcriptome may provide insights into the mechanisms underlying cellular functions and precursor responses in the jejunum. Thus, the natural next step is to study gene expression in jejunal tissue under a diet with and without carnosine synthesis precursors.
